# Fragment-based discovery of the first nonpeptidyl inhibitor of an S46 family peptidase

**DOI:** 10.1038/s41598-019-49984-3

**Published:** 2019-09-19

**Authors:** Yasumitsu Sakamoto, Yoshiyuki Suzuki, Akihiro Nakamura, Yurie Watanabe, Mizuki Sekiya, Saori Roppongi, Chisato Kushibiki, Ippei Iizuka, Osamu Tani, Hitoshi Sakashita, Koji Inaka, Hiroaki Tanaka, Mitsugu Yamada, Kazunori Ohta, Nobuyuki Honma, Yosuke Shida, Wataru Ogasawara, Mayumi Nakanishi-Matsui, Takamasa Nonaka, Hiroaki Gouda, Nobutada Tanaka

**Affiliations:** 1 School of Pharmacy, Iwate Medical University, 1-1-1 Idaidori, Yahaba, Iwate 028-3694 Japan; 2 Department of Bioengineering, Nagaoka University of Technology, 1603-1 Kamitomioka, Nagaoka, Niigata 940-2188 Japan; 3 National Institute of Technology, Nagaoka College, 888 Nishikatakai, Nagaoka, Niigata 940-8532 Japan; 4 School of Pharmacy, Showa University, 1-5-8 Hatanodai, Shinagawa-ku, Tokyo 142-8555 Japan; 5 Biomedical Research Institute, National Institute of Advanced Industrial Science and Technology (AIST), 1-1-1 Higashi, Tsukuba, Ibaraki 305-8566 Japan; 6 Maruwa Foods and Biosciences Inc., 170-1 Tsutsui-cho, Yamatokoriyama, Nara 639-1123 Japan; 7 Confocal Science Inc., 2-12-2 Iwamoto-cho, Chiyoda-ku, Tokyo 101-0032 Japan; 8 Japan Aerospace Exploration Agency (JAXA), 2-1-1 Sengen, Tsukuba, Ibaraki 305-8505 Japan; 9 Center for Molecular Analysis, Showa University, 1-5-8 Hatanodai, Shinagawa-ku, Tokyo 142-8555 Japan

**Keywords:** Virtual screening, Proteases, X-ray crystallography

## Abstract

Antimicrobial resistance is a global public threat and raises the need for development of new antibiotics with a novel mode of action. The dipeptidyl peptidase 11 from *Porphyromonas gingivalis* (PgDPP11) belongs to a new class of serine peptidases, family S46. Because S46 peptidases are not found in mammals, these enzymes are attractive targets for novel antibiotics. However, potent and selective inhibitors of these peptidases have not been developed to date. In this study, a high-resolution crystal structure analysis of PgDPP11 using a space-grown crystal enabled us to identify the binding of citrate ion, which could be regarded as a lead fragment mimicking the binding of a substrate peptide with acidic amino acids, in the S1 subsite. The citrate-based pharmacophore was utilized for *in silico* inhibitor screening. The screening resulted in an active compound SH-5, the first nonpeptidyl inhibitor of S46 peptidases. SH-5 and a lipophilic analog of SH-5 showed a dose-dependent inhibitory effect against the growth of *P*. *gingivalis*. The binding mode of SH-5 was confirmed by crystal structure analysis. Thus, these compounds could be lead structures for the development of selective inhibitors of PgDPP11.

## Introduction

Periodontitis is a bacterially induced inflammatory disease that destroys tooth-supporting tissues, eventually leading to tooth loss^1^. Periodontitis is one of the most common diseases worldwide, and as of 2017, chronic periodontitis affects approximately 796 million people annually^2^. It has been reported that periodontitis is associated with various systemic diseases, such as diabetes and cardiovascular disease^3^, preterm and low-weight births^4^, Alzheimer’s disease^5,6^, cancers^7^, respiratory diseases^8^, and rheumatoid arthritis^9^. Although as many as 700 different bacterial species can be present in the oral cavity^10^, only a small percentage of these species have pathogenic potential. *Porphyromonas gingivalis*, a gram-negative black-pigmented anaerobic bacterium, is a major pathogen associated with the chronic form of periodontitis^11^. Because *P*. *gingivalis* is an asaccharolytic bacterium that gains its metabolic energy by fermenting amino acids instead of carbohydrates, *P*. *gingivalis* is known to be the most highly proteolytic bacterium colonizing the oral cavity and produces several types of peptidases: cysteine peptidases (gingipains), collagenases, and di- or tripeptidyl peptidases^12,13^. Gingipains—Arg-gingipain A, Arg-gingipain B and Lys-gingipain—are responsible for the extracellular and cell-bound proteolytic activities and are implicated as major virulence factors of *P*. *gingivalis*^14–16^. Extracellular oligopeptides produced by gingipains are converted to di- or tripeptides in the periplasm by several peptidases^13^. These di- or tripeptides are incorporated into the cytoplasm via oligopeptide transporters^17^. Because *P*. *gingivalis* utilizes di- and tripeptides, instead of single amino acids, as sources of energy, peptidases that provide di- and tripeptides in the periplasm are essential for the metabolic activity of the bacterium^18,19^. *P*. *gingivalis* dipeptidyl peptidase 4 (PgDPP4) is reported to act in concert with collagenases to produce short peptides^20,21^. Recently, the novel DPPs DPP5 (PgDPP5), DPP7 (PgDPP7) and DPP11 (PgDPP11) were identified in *P*. *gingivalis*^22–24^. Among these DPPs, PgDPP7 and PgDPP11 have been assigned to a novel type of serine peptidase family, S46, in clan PA^22,23^. Because S46 peptidases are widely distributed in anaerobic gram-negative species in the genera *Bacteroides*, *Parabacteroides*, and *Porphyromonas* but not in mammals^23,25^, these peptidases are ideal targets for novel antibiotics. PgDPP11 exhibits a strict substrate specificity for acidic residues (Asp/Glu) at the P1 position (NH_2_-P2-P1-P1’-P2’-…, where the P1-P1’ bond is the scissile bond)^26^, whereas PgDPP7 exhibits a broad substrate specificity for both aliphatic and aromatic residues at the P1 position. It is thought that PgDPP11 plays an important role in the metabolism of *P*. *gingivalis* by degrading polypeptides carrying Asp and Glu, because Asx (Asp and Asn) and Glx (Glu and Gln) are the most abundantly utilized amino acids in this bacterium^18,19^. Nemoto and coworkers showed that a *dpp11*-knockout strain lost most of its Asp/Glu-dependent DPP activity, and growth of the strain was significantly retarded^23^.

Recently, crystal structures of PgDPP11 were solved^27,28^, and those of *Porphyromonas endodontalis* DPP11 (PeDPP11) have also been reported^28^. PgDPP11 is a homodimer, and each subunit contains a peptidase domain, including a double β-barrel fold that is characteristic of the chymotrypsin superfamily^29,30^, as well as an unusual α-helical domain that regulates the exopeptidase activity of PgDPP11. The structures of PgDPP11 clearly showed that the residues directly involved in recognition of the N-terminal amino group of the substrate peptide are Asn218, Trp219, Asn333 and Asp672, and the catalytic triad is His85-Asp227-Ser655^27^. Biochemical studies and crystal structure analyses revealed that Arg673 in the S1 subsite of PgDPP11 is a crucial residue for the strict Asp/Glu P1 specificity of PgDPP11^23,27,31^. Arg673 of PgDPP11 is replaced by Gly666 in PgDPP7. For dipeptidyl peptidase BII (DAP BII, a DPP7 homolog) from the gram-negative aerobic bacterium *Pseudoxanthomonas mexicana* WO24, the corresponding residue in the S1 subsite is Gly675, and the S1 subsite of DAP BII is deep enough to accommodate an aromatic P1 residue^32^. Analogous to PgDPP7, DAP BII exhibits a broad substrate specificity for both aliphatic and aromatic residues at the P1 position^27^. Because the overall structure, the molecular basis of the exopeptidase activity, the catalytic mechanism, and the substrate recognition mechanisms of S46 peptidases have been elucidated by crystal structure analyses of DAP BII and PgDPP11, structure-based inhibitor design for PgDPP11 for the development of antibacterial agents has become possible. However, potent and selective inhibitors of S46 peptidases, both peptidyl and nonpeptidyl, have not been developed to date.

In this study, we determined a crystal structure of PgDPP11 in complex with citrate ions at a 1.50 Å resolution using a space-grown crystal. The bound citrate ion, a potassium ion, and a water molecule in the S1 subsite of PgDPP11 were regarded to mimic the binding of an acidic amino acid and were utilized as a pharmacophore for an *in silico* inhibitor screening. The screening resulted in the first nonpeptidyl inhibitor of S46 peptidases, SH-5 (2-[(2-aminoethyl)amino]-5-nitrobenzoic acid, C_9_H_11_N_3_O_4_). The binding mode of SH-5 was confirmed by crystal structure analysis at a 2.39 Å resolution. The hit compound SH-5 and a related compound identified and evaluated in the present study may represent novel starting points for further rational design of potent inhibitors against PgDPP11.

## Results

### Crystal structure of PgDPP11 complexed with citrate ions

**Figure 1:**
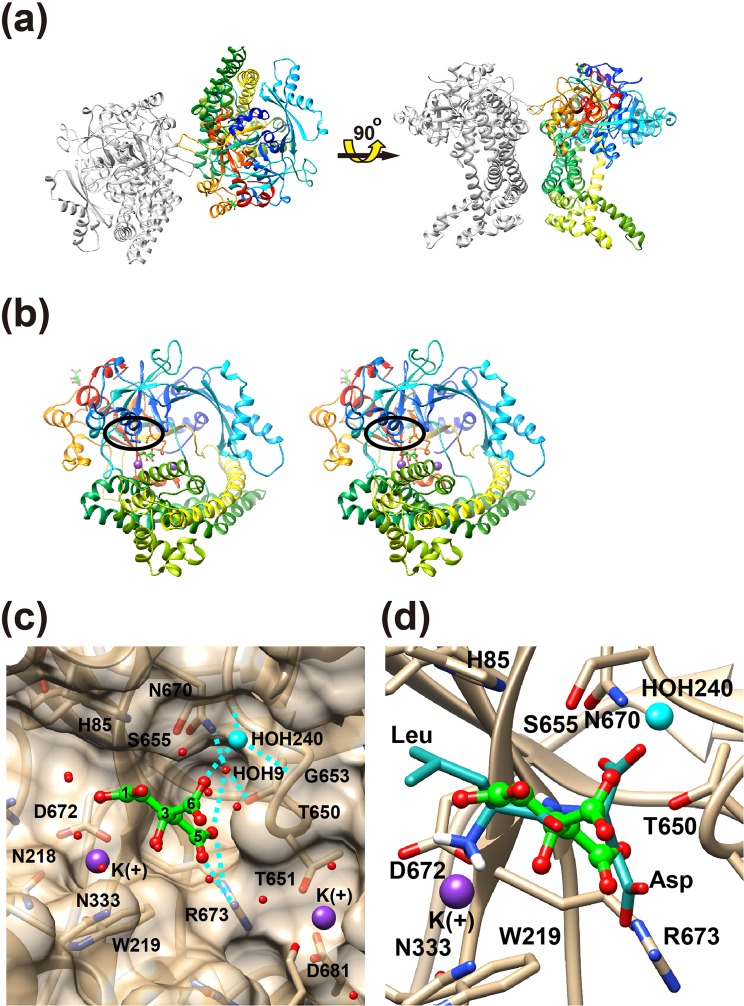
Three-dimensional structure of the citrate complex of PgDPP11. (**a**) Dimeric structure of PgDPP11. One subunit is colored in rainbow colors from the N-terminus (blue) to the C-terminus (red), and the other is colored gray. (**b**) A stereo diagram showing the PgDPP11 subunit. The catalytic domain is colored in blue to cyan and orange to red. The α-helical domain is colored in yellow to green. The catalytic triad “Asp227-His85-Ser655” is marked by an ellipsoid. The bound citrate (green) and potassium (purple) ions are shown in ball-and-stick and sphere models, respectively. (**c**) The mode of citrate ion binding in the S1 subsite of PgDPP11. Possible hydrogen bonds and salt bridges are shown as dashed lines. (**d**) Comparison of the present crystal structure with a dipeptide (Leu-Asp, blue) docking model of PgDPP11. The bound water molecules except for HOH240 were removed for clarity.

**Figure 2:**
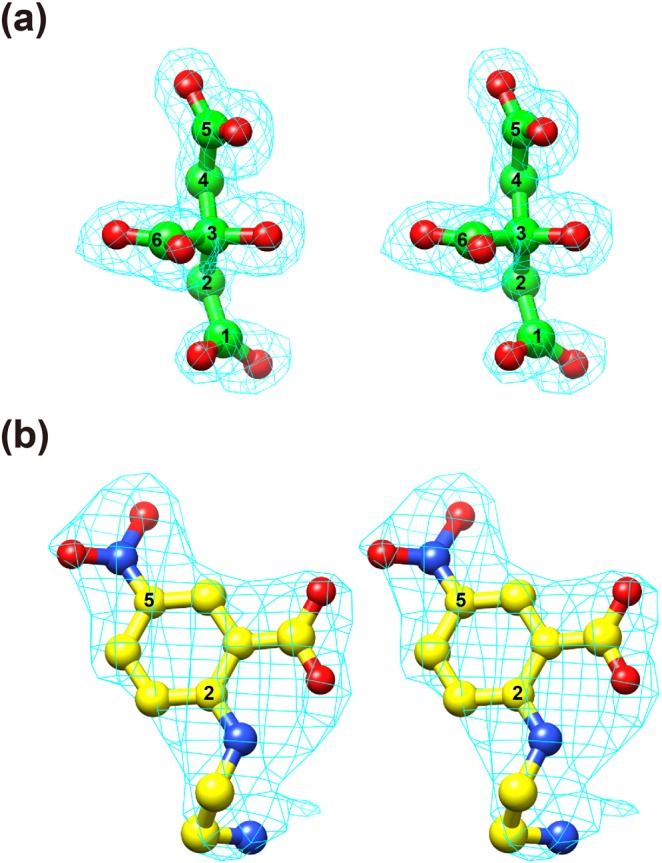
Stereodiagrams showing weighted *m*|Fo|-*D*|Fc| omit maps of the bound ligand molecule in the S1 subsite of PgDPP11. The contour levels are 4.0 σ (cyan). (**a**) Citrate ion at a 1.50-Å resolution. (**b**) SH-5 at a 2.39-Å resolution.

The PgDPP11 enzyme forms a homodimer, with each subunit consisting of 699 amino acid residues (Asp22-Pro720) and a molecular weight of approximately 160 kDa (Fig. 1a). The crystal structure of PgDPP11 in complex with citrate and potassium ions was determined at a 1.50 Å resolution by analyzing a space-grown crystal. The final *R* and *R*_free_ values were 0.170 and 0.184, respectively, at a 1.50 Å resolution (Tables 1 and 2). A protomer of PgDPP11 is situated in the asymmetric unit (Fig. 1b). Two protomers of PgDPP11 are related by a crystallographic two-fold axis of the *C*222_1_ crystal and form a dimer (Fig. 1a). The protomer of PgDPP11 consists of two domains (Fig. 1b). One domain, containing the double β–barrel fold characteristic of the chymotrypsin superfamily and harboring the Asp-His-Ser catalytic triad, is responsible for catalysis; the other, the α-helical domain, is a regulatory domain that is necessary for exopeptidase activity. High-resolution diffraction data obtained from a space-grown crystal enabled us to identify two potassium ion-binding sites and two citrate ion-binding sites in the protomer of PgDPP11 because the present crystallization conditions contained 0.20 M tri-potassium citrate in the reservoir solution. In the previous crystal structure analysis at a 1.66 Å resolution^27^, no citrate ion was identified in the protomer of PgDPP11, while two potassium ions were identified in the same binding sites as those observed in this study. These results may be caused by some differences in the quality of the diffraction data between the previous study at a 1.66 Å resolution and the present study at a 1.50 Å resolution. In this study, the citrate ion was identified in the S1 subsite of PgDPP11, and the other was identified on the surface of the molecule and found to interact with the side chain of Arg536. A representative electron density map of the bound citrate ion in the S1 subsite is shown in Fig. 2.

**Table 1:** Data collection statistics for PgDPP11.

Data set	Citrate complex	SH-5 complex
Facility	Photon Factory	Photon Factory
Beamline	BL17A	BL17A
Wavelength (Å)	0.98	0.98
Detector	Pilatus 6 M	Pilatus 6 M
Crystal-to-detector distance (mm)	395.1	485.68
Rotation angle per image (°)	0.05	0.2
Total rotation range (°)	190	190
Exposure time per image (sec)	0.25	1
Space group	*C*222_1_	*P*2_1_2_1_2_1_
Cell dimensions		
*a* (Å)	99.13	102.33
*b* (Å)	103.35	116.96
*c* (Å)	176.52	148.2
α (°)	90	90
β (°)	90	90
γ (°)	90	90
Number of molecules per ASU	1	2
Mosaicity (°)	0.206	0.093
Resolution (Å)	49.56–1.50	39.62–2,39
(outer shell)	(1.53–1.50)	(2.44–2.39)
No. of observed reflections	941,940	487,361
	−31,223	−32,276
No. of unique reflections	144,097	70,931
	−7,050	−4,484
Completeness (%)	99.9 (99.3)	99.9 (99.7)
Redundancy	6.5 (4.4)	6.9 (7.2)
*I*/σ_(*I*)_	17.7 (2.1)	9.2 (2.1)
CC_half_	0.999 (0.688)	0.990 (0.680)
*R*_merge_ (*I*)	0.045 (0.606)	0.146 (1.222)
*R*_meas_ (*I*)	0.053 (0.764)	0.171 (1.419)
*R*_pim_ (*I*)	0.020 (0.352)	0.088 (0.715)
Wilson *B*-factor (Å^2^)	18.6	30.9

**Table 2:** Refinement statistics for PgDPP11.

Data set	Citrate	SH-5
PDB ID	6JTB	6JTC
Resolution range (Å)	49.56–1.50	39.62–2.39
Completeness (%)	99.84	99.75
No. of reflections		
working set	136,886	67,329
test set	7,155	3,696
*R*-factor	0.169	0.201
Free *R*-factor	0.192	0.242
No. of protein atoms (avg. *B*-factors (Å2))	5,620	11,376
	−24.3	−45
No. of ligand atoms (avg. *B*-factors (Å2))	26	32
	(2 × 13)	(2 × 16)
	−27.2	−49.4
No. of glycerol atoms (avg. *B*-factors (Å2))	30	0
	(5 × 6)	
	−36.7	
No. of water molecules (avg. *B*-factors (Å2))	845	76
	−34.1	−34.4
Ramachandran plot statistics		
favored (%)	684 (98.1)	1,345 (96.5)
allowed (%)	13 (1.9)	46 (3.3)
outlier (%)	0 (0.0)	3 (0.2)
RMSD		
bonds (Å)	0.014	0.01
angles (°)	1.678	1.442

### The active site of PgDPP11

The catalytic triad of PgDPP11 is Asp227-His85-Ser655, and the oxyanion hole is formed by the main-chain amide nitrogen atoms of Ser655 and Gly653^27^. The N-terminal amino group recognition residues essential for the exopeptidase activity of PgDPP11 are Asn218, Trp219, and Asp672 from the catalytic domain and Asn333 from the α-helical domain^27^. The S1 subsite of PgDPP11 consists of His649, Thr650, Thr651, Gly652, Asn670, Arg673, Gly677, Gly680, Asp681, and Ser691 and is mainly composed of hydrophilic residues^27^. Arg673, a crucial residue for the Asp/Glu specificity of PgDPP11^23,27,31^, is located on the wall of the S1 subsite (Fig. 1c), and the corresponding residue in DAP BII is Gly675. The most remarkable observation in the present crystallographic study is that one citrate ion was accommodated in the S1 subsite. A distal carboxy group (O3C5O4) of the citrate ion forms bifurcated salt bridges with the side chain of Arg673 (O4(citrate)–NE(Arg673): 2.8 Å and O3(citrate)–NH2(Arg673): 2.8 Å) (Fig. 1c) and is found to mimic the binding mode of an acidic (Asp/Glu) side chain of the P1 residue of a substrate peptide. The other distal carboxy group (O1C1O2) at the opposite end is exposed to solvent. A water molecule that is hydrogen bonded to the central carboxy group (O5C6O6) of the citrate (O6(citrate)–O(HOH240): 2.9 Å) is accommodated in the oxyanion hole of PgDPP11 (N(Gly653)–O(HOH240): 2.9 Å and N(Ser655)–O(HOH240): 3.0 Å) (Fig. 1c). In addition, a potassium ion was identified in the P2 residue-binding site of PgDPP11 and found to mimic the binding mode of the positively charged N-terminus of the P2 position of the substrate peptide. The central hydroxy group of the citrate forms an ion-dipole interaction with the potassium ion (O7(citrate)–K: 3.0 Å) or forms an intramolecular hydrogen bond with the carboxy group of the citrate. The other potassium ion was found at the bottom of the S1 subsite and appeared to be trapped by an electrostatic interaction with the side chain of Asp681. Comparison the binding mode of the citrate ion described above and a dipeptide docking model of PgDPP11^27^ indicates that the potassium ion, the carboxy group of citrate and the water molecule (HOH240) mimic the binding sites of the N-terminus, the acidic P1 side chain and the carbonyl group of the P1 residue of the bound peptide, respectively (Fig. 1d).

### *In silico* screening

**Figure 3:**
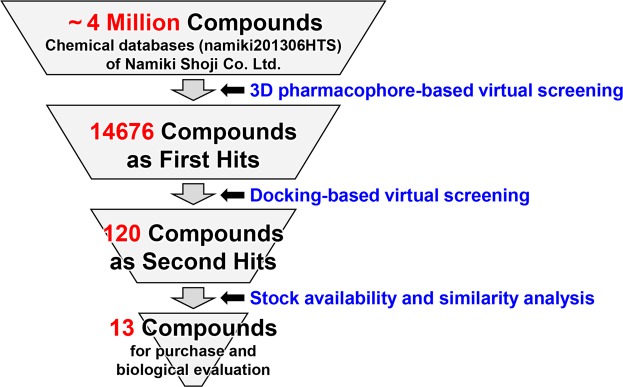
Flowchart of multifilter virtual screening.

We executed a multifilter virtual screening protocol to explore candidate compounds for novel PgDPP11 inhibitors (Fig. 3).

**Figure 4:**
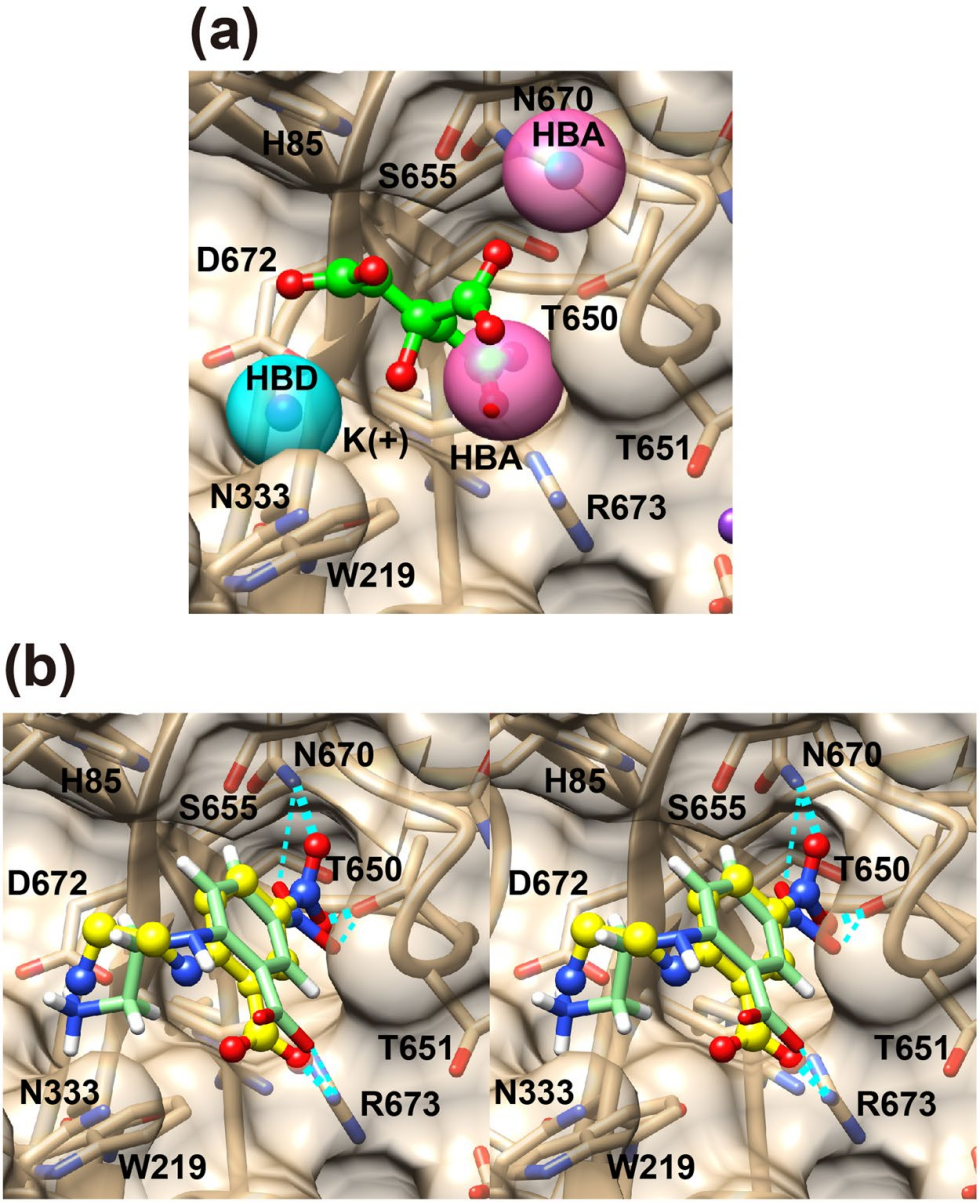
Mode of SH-5 binding in the S1 subsite of PgDPP11. The *in silico* model is shown as a stick model with hydrogens. (**a**) A 3D pharmacophore model for the first-stage screening. Hydrogen bond donor (HBD) and acceptor (HBA) features are shown as cyan and magenta spheres, respectively. (**b**) A stereo diagram showing *in silico* docking model (light green) based on MM-GBSA scoring and present crystal structure (yellow) at a 2.39 Å resolution. Possible hydrogen bonds and salt bridges are shown as thick and thin dashed lines for the crystal structure and docking model, respectively.

In the first stage, we performed three-dimensional (3D) pharmacophore-based virtual screening. We generated a 3D pharmacophore model (Fig. 4a) based on a distal carboxy group of citrate, a water molecule bound to the central carboxy group of citrate (HOH240), and a potassium (K^+^) ion in a space-grown crystal-derived high-resolution crystal structure of the PgDPP11/citrate complex using the Unity module implemented in SYBYL-X Suite (Certara USA Inc., Princeton, NJ, USA). We defined two hydrogen bond acceptor (HBA) features (Fig. 4a, magenta spheres) at the centroid of two oxygen atoms of the distal carboxy group of citrate and at the position of the oxygen atom of HOH240. In addition, we defined one hydrogen bond donor (HBD) feature (Fig. 4a, cyan sphere) at the position of the K^+^ ion, because this position was suggested to correspond to the positively charged N-terminus of the P2 position of the substrate peptide. Therefore, the pharmacophore model includes a total of three features, consisting of two HBA features and one HBD feature. The pharmacophore model was used as a 3D structural query for retrieving compounds from the chemical database (namiki201306HTS) of Namiki Shoji Co., Ltd. (Tokyo, Japan) using the Unity module. The 3D coordinates of each molecule were generated with the CONCORD module of SYBYL, and the flex search protocol was performed to screen the database. We used a surface volume constraint based on a MOLCAD surface of the active site of PgDPP11. We extracted compounds with molecular weights less than 300. As a result, we obtained 14,676 compounds as “first hits” from the model. The first hits were subsequently screened using protein structure-based virtual molecular docking (Fig. 3).

In the second stage, molecular docking was performed using Schrödinger Suite 2013-2 (Schrödinger, LLC, New York, NY, USA). First, the 3D structures of 14,676 hits were constructed using the LigPrep 2.7 program, and the protonation states of these structures were predicted using the Epik 2.5 program. The resulting conformers were used in the following docking calculations. The crystal structure of PgDPP11 described above was used as a receptor for docking. The crystal structure was minimized using force-field OPLS 2005 through the Protein Preparation Wizard in Maestro 9.5. We removed citrate ions, potassium ions, and H_2_O molecules before docking calculations. A docking grid that was 20 × 20 × 20 Å cube was generated with default settings using the cocrystallized citrate ion in the active site as the centroid while ensuring that the grid box was large enough to cover the entire active site of PgDPP11. We used a virtual screening workflow implemented in Schrödinger Suite 2013-2. Then, first-stage docking was performed in HTVS mode using GLIDE version 6.0 of Schrödinger Suite 2013-2, and the top 10% of the poses were subjected to second-stage docking in SP mode. In the first and second docking calculations, we used hydrogen bond constraints, i.e., docking poses were generated to form hydrogen bonds with at least one nitrogen atom (NE and/or NH2) of the side chain of Arg673 of PgDPP11, which is known to form hydrogen bonds with the carboxy group of the P1 residue of the substrate peptide (Fig. 1d). Finally, binding free energy (ΔG_binds_) values for the top 10% of the poses derived from second-stage docking were estimated by the molecular mechanics generalized-born surface area (MM-GBSA) method^33,34^ using the Prime 3.3 program. We extracted 120 compounds with ΔG_bind_ values lower than –30.00 kcal/mol as “second hits” (Fig. 3).

In the third stage, we first removed hazardous substances from the second hits and checked stock availability to select 63 compounds that were available for purchase at that time. The 63 selected compounds were finally clustered using similarity analysis based on their two-dimensional structural fingerprints^35,36^ to reduce the number of virtual hits with which to proceed. Clustering was performed with Canvas 1.7 of Schrödinger Suite 2013-2 (Fig. 3). As a result, we selected 15 compounds representing the cluster, 13 of which (Fig. S1, named compounds SH-1 to SH-13) were purchased to perform the following biological evaluation. One of the two unpurchased compounds was a dipeptide compound, and the other was not available for purchase at a later date.

### Experimental evaluation of *in silico* candidate compounds

**Figure 5:**
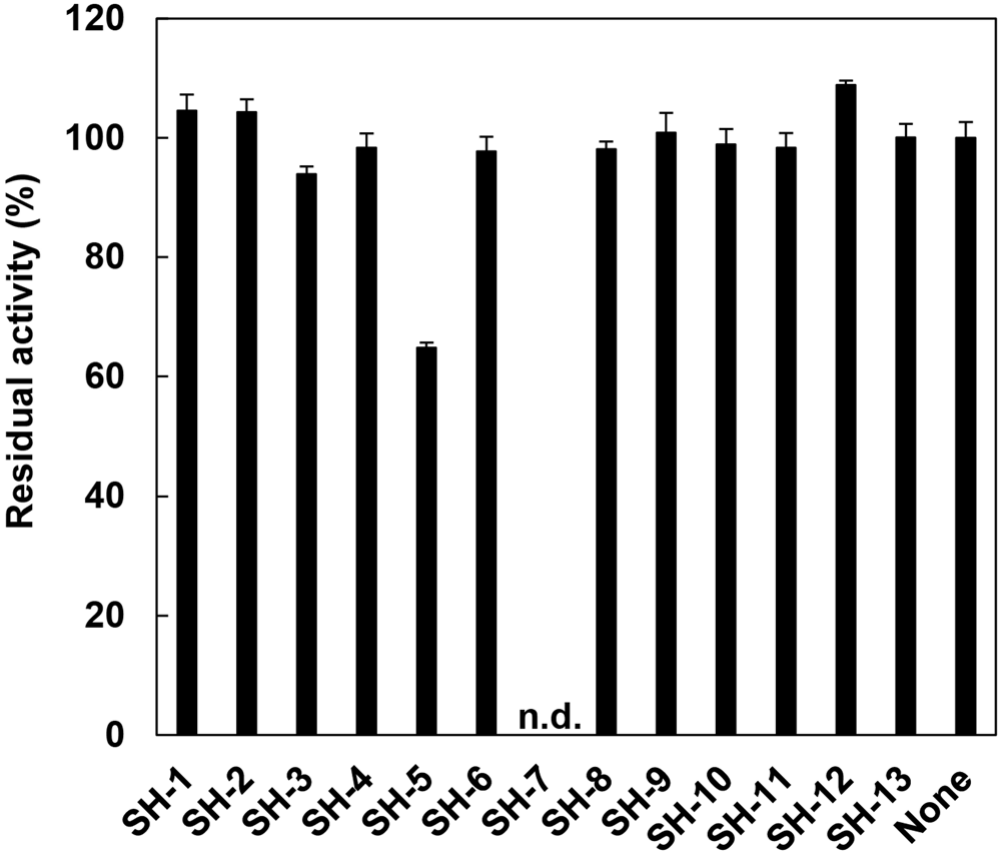
Inhibitory effects of 13 candidate compounds obtained by multifilter *in silico* screening. Residual activities (100% activity = activity without inhibitor, 3.15 ± 0.08 U/mg) were measured under conditions where the concentrations of the synthetic substrate Leu-Asp-MCA and inhibitors were 100 µM. The inhibitory effect of SH-7 was not detected (n.d.), because SH-7 is a fluorescent compound and exhibits excitation (Ex) and emission (Em) wavelengths of 370 and 450 nm, respectively, similar to those of the synthetic substrate LD-MCA (Ex/Em = 355/460 nm). Standard deviations were obtained from three independent experiments.

We evaluated the inhibitory effects of the 13 commercially available candidate compounds obtained by the *in silico* screening described above against the enzymatic activity of PgDPP11 on the synthetic substrate Leu-Asp-MCA (4-methylcoumaryl-7-amide) (Fig. 5). Each compound was first tested at a concentration of 100 μM. Among the 13 candidate compounds, only SH-5 (2-[(2-aminoethyl)amino]-5-nitrobenzoic acid, C_9_H_11_N_3_O_4_) showed a significant inhibitory effect (>30%) against PgDPP11 (Fig. 5). Dose-response curves (1000, 500, 250, 125, 62.5, 31.3 and 15.6 μM) were generated for SH-5, and the IC_50_ and *K*_i_ values of SH-5 were estimated to be approximately 90.1 and 8.45 μM, respectively (Table 3). Because SH-5 is regarded to be a fragment (Mw: 225.2), the hit exhibited moderate activity compared to lead-like inhibitors but is an adequate starting point for optimization based on the ligand efficiency (LE, roughly calculated as LE = 1.37pIC_50_/HA) of 0.346^37,38^.

**Table 3:** Inhibitory effects of SH-5 and NPPB against DPPs.

Enzyme	SH-5		NPPB	
	Residual activity (%)	*K*_i_ (µM)	Residual activity (%)	*K*_i_ (µM)
PgDPP11	62.9 ± 0.1	8.45 ± 0.38	70.3 ± 0.7	15.0 ± 0.7
SmDPP11	48.0 ± 1.1	24.9 ± 0.5	67.7 ± 0.6	84.8 ± 3.1
PgDPP7	54.4 ± 0.7	52.2 ± 3.3	159 ± 5	ND
SmDPP7	72.8 ± 0.7	36.6 ± 1.3	119.0 ± 0.3	ND

^*^ND: not determined due to low inhibitory effect at present substrate concentration (100 µM). Residual activities (100% activity = activity without inhibitor) were measured under the conditions where the concentrations of inhibitor and synthetic substrate were 100 µM.

### *In silico* analyses of the binding mode of SH-5 with PgDPP11

To identify the inhibition mechanisms of the hit compound SH-5 at an atomic level, the interaction mode between SH-5 and PgDPP11 was examined by using a combination of the molecular docking calculation and MM-GBSA free energy analysis, as detailed in the Methods. The resulting interaction model of SH-5 with PgDPP11 obtained by our procedure is shown in Fig. 4b. SH-5 was suggested to bind at a similar location as that at which citrate ion binds with PgDPP11. The carboxy group of SH-5 was found to form an ionic hydrogen bond with Arg673 of PgDPP11, mimicking the distal carboxy group of the citrate ion. The nitro group of SH-5 was also suggested to form two hydrogen bonds with the hydroxy group of Thr650 and the carboxamide group of Asn670 of PgDPP11. The location of this nitro group was corresponded to HOH9 in the crystal structure, which forms a water-mediated hydrogen bond network between the citrate ion and PgDPP11 (Fig. 1c). The location of the tertiary amine of SH-5 also corresponded to the potassium ion in the crystal structure. This tertiary amine was found to form a hydrogen bond with Asn218 and an electrostatic interaction with Asp672. In total, SH5 seems to bind with PgDPP11 in a similar fashion as citrate ion, potassium ion, and HOH9.

### Experimental verification of the *in silico*-predicted binding mode of SH-5

To verify the *in silico*-predicted binding mode of SH-5 in the active site of PgDPP11 (Fig. 4b), we tried to obtain crystals of PgDPP11 in complex with SH-5. The best crystal was obtained using a counterdiffusion crystallization method under a microgravity environment in the International Space Station (ISS). The crystal structure of PgDPP11 in complex with SH-5 was determined at a 2.39 Å resolution by analyzing the space-grown crystal. The final *R* and *R*_free_ values were 0.201 and 0.237, respectively, at a 2.39 Å resolution (Tables 1 and 2). Two protomers of PgDPP11 were involved in an asymmetric unit of the *P*2_1_2_1_2_1_ crystal and formed a dimer. As expected based on our *in silico* study, one molecule of SH-5 was observed in each of the S1 subsites of the PgDPP11 subunits (Fig. 2). The crystal structure (Fig. 4b, yellow) revealed that the *in silico* docking model based on MM-GBSA scoring (Fig. 4b, light green) properly represented the crystal structure.

### Inhibitory effects of SH-5 analog

Five additional compounds (Fig. S2) belonging to the same cluster as SH-5 in a similarity analysis based on two-dimensional structural fingerprints^35,36^ were also evaluated. Of the five compounds, only SH-5_3 (2-(4-carbamoyl-2-nitrophenyl)sulfanylacetic acid, C_9_H_8_N_2_O_5_S, Mw: 256.2) showed a significant but relatively weak inhibitory effect against PgDPP11, and the IC_50_ value of SH-5_3 was estimated to be approximately 1065 μM. In addition to the five additional compounds, the inhibitory effect of a lipophilic structural analog of SH-5 (5-nitro-2-(3-phenylpropylamino)benzoic acid, C_16_H_16_N_2_O_4_, hereafter NPPB, Mw: 300.31, Fig. S2) was evaluated. The *K*_i_ value of NPPB was estimated to be approximately 15.0 μM (Table 3). Thus, a common building block of SH-5 and related compounds, i.e., the nitrobenzoic acid group, appears to be a key feature for the interaction with the active site of PgDPP11.

### Biological evaluation

**Figure 6:**
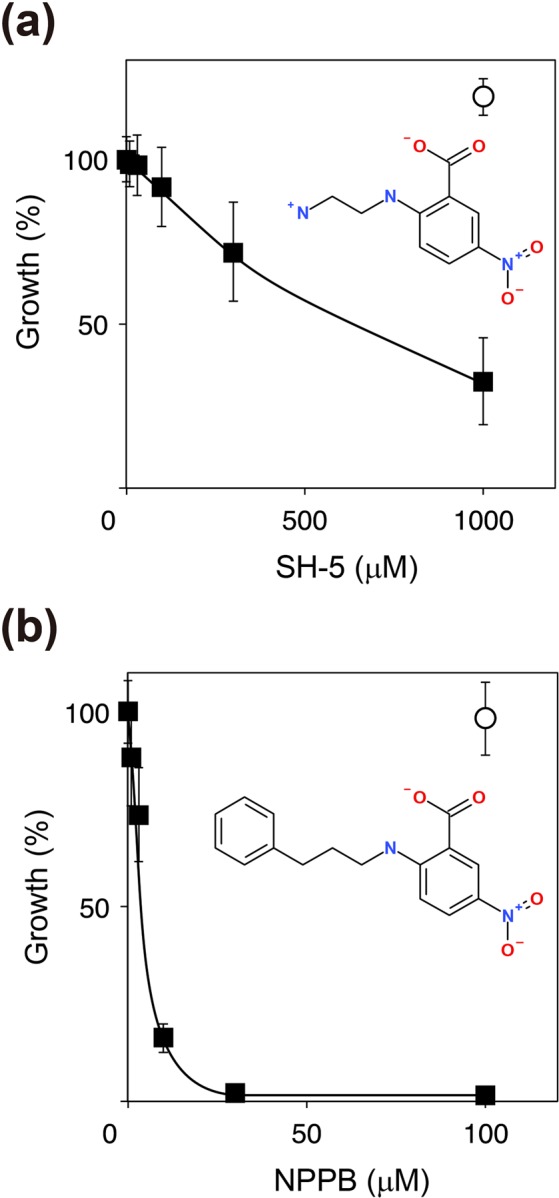
Biological evaluations of the antigrowth activity of SH-5 and the lipophilic analog NPPB against the *P*. *gingivalis* strain W83 (closed squares) and *E*. *coli* strain K12 (open circles). (**a**) SH-5. (**b**) NPPB.

The inhibitory effects of SH-5 and the lipophilic analog NPPB against growth of *P*. *gingivalis* strain W83 were evaluated by monitoring optical density at 600 nm (Fig. 6, closed squares). SH-5 showed a dose-dependent inhibitory effect against the growth of *P*. *gingivalis* (Fig. 6a). However, a relatively high concentration of the inhibitor was required (IC_50_ value of 687 μM). Interestingly, the lipophilic analog of SH-5, NPPB, showed a relatively strong inhibitory effect (IC_50_ value of 6 μM) (Fig. 6b). The maximal percent inhibition values for SH-5 and NPPB were 68% at 1 mM and 99% at 100 μM, respectively. Because *E*. *coli* does not have S46 peptidases, the inhibitory effects of the two compounds against the growth of *E*. *coli* strain K12 were also evaluated. The results showed that the two compounds were not toxic toward *E*. *coli* at the concentrations used for the inhibition of *P*. *gingivalis* growth (Fig. 6, open circles). The biological evaluation presented here shows that (i) NPPB has a stronger inhibitory effect against the growth of *P*. *gingivalis* than SH-5; (ii) a bacterial species selectivity, i.e., the compound effective against *P*. *gingivalis* but not *E*. *coli*, does exist in the growth inhibition by SH-5 and the lipophilic analog NPPB; and (iii) the growth inhibition is assumed to be largely attributable to inhibition of PgDPP11.

### Specificity of SH-5 against S46 peptidases

To examine whether SH-5 is a universal inhibitor of S46 peptidases or is specific for PgDPP11, we evaluated the inhibitory effects of SH-5 against PgDPP7. In addition, the inhibitory effects of SH-5 against DPP7 and DPP11 from *Stenotrophomonas maltophilia* (SmDPP7 and SmDPP11, respectively) were also examined as another reference set of S46 peptidases in the same organism. As expected, NPPB showed significant inhibitory effects against both PgDPP11 and SmDPP11, whereas the compound was inactive against both PgDPP7 and SmDPP7 (Table 3). Interestingly, SH-5 exhibited significant inhibitory effects against the DPP7s as well as the DPP11s (Table 3). Thus, NPPB exhibits clear target specificity against DPP11s over DPP7s, whereas SH-5 shows a slight preference for DPP11s over DPP7s.

## Discussion

**Figure 7:**
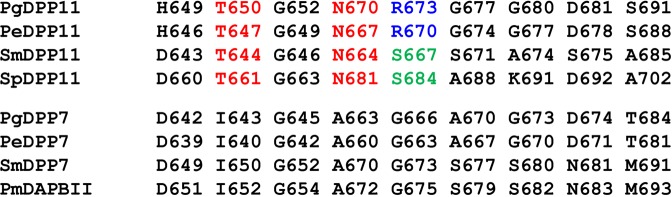
Comparison of the residues in the S1 subsite of DPP7-type and DPP11-type S46 peptidases. Residues that form hydrogen bonds with the nitro group of SH-5, Thr650 and Asn670 in PgDPP11 (Fig. 4b) and corresponding residues conserved in other DPP11s are coloured red. Arg673, which is crucial for the strict Asp/Glu specificity of PgDPP11, is shown in blue. The arginine residue is also conserved in PeDPP11 (Arg670) but is replaced by a serine residue (green) in SmDPP11 (Ser667) and SpDPP11 (Ser684).

In this study, pharmacophore-based *in silico* screening resulted in the discovery of SH-5, the first nonpeptidyl inhibitor of S46 peptidases. The binding mode of SH-5 was confirmed by X-ray crystallography. The *K*_i_ vales of SH-5 and the lipophilic analog NPPB against PgDPP11 were estimated to be 8.45 and 15.0 μM, respectively (Table 3). For the substrate specificity of S46 peptidases, the peptidases were roughly grouped into two groups according to their specificity/preference of the P1 residue of the substrate peptide (NH_2_-P2-P1-P1’-P2’-…, where the P1-P1’ bond is the scissile bond)^26^: DPP11s exhibit a strict substrate specificity for acidic residues (Asp/Glu) at the P1 position, whereas DPP7s exhibit a broad substrate specificity for both aliphatic and aromatic residues at the P1 position. Our data showed that NPPB and SH-5 exhibit significant inhibitory effects against DPP11s over DPP7s, though SH-5 shows a slight preference (Table 3). The crystal structure of PgDPP11 complexed with SH-5 (Fig. 4b, yellow) showed that the carboxy group of SH-5 forms an electrostatic interaction with the side chain of Arg673, a crucial residue for the Asp/Glu specificity of PgDPP11^23,27,31^. This experimental observation is consistent with the previously obtained *in silico* docking model based on MM-GBSA scoring (Fig. 4b, light green). The crystal structure of the SH-5 complex also revealed that the 5-nitro group of SH-5 is involved in a hydrogen bond network with the side chains of Thr650 and Asn670. An amino acid sequence comparison of residues in the S1 subsite of typical S46 peptidases (Fig. 7) shows that Thr650 and Asn670 in PgDPP11 are well conserved among DPP11s but are replaced by hydrophobic residues, namely, Ile and Ala, respectively, in DPP7s. This result indicates that the hydrogen bond network between Thr650 and Asn670 exists specifically in DPP11s (but not in DPP7s), and the nitro group of SH-5 (and its analog) is also important for the selectivity of this type of inhibitor against DPP11s over DPP7s. For the selectivity, the electrostatic interaction between Arg673 and the carboxy group of SH-5 appears to be more dominant than the hydrogen bond network involving Thr650, Asn670 and the nitro group of SH-5 (Fig. 4b), because Arg673 is replaced by Ser667 in SmDPP11 (Fig. 7). Indeed, the inhibitory effect of SH-5 against SmDPP11 (*K*_i_ value of 24.9 μM) is slightly higher than that against SmDPP7 (*K*_i_ value of 36.6 μM), whereas the inhibitory effect against PgDPP11 (*K*_i_ value of 8.45 μM) is significantly higher than that against PgDPP7 (*K*_i_ value of 52.2 μM) (Table 3).

The structure-activity relationship of SH-5 and its lipophilic analog NPPB is quite reasonable. As shown in Fig. 6, the antigrowth activity of NPPB against the *P*. *gingivalis* strain W83 is superior than that of SH-5, while the inhibitory effect of NPPB against the enzymatic activity of PgDPP11 is comparable to (or weaker than) that of SH-5 (Table 3). This observation is explained by the clear difference in the lipophilicity of the compounds. The aminoethyl group of SH-5 is replaced by a phenylpropyl group in NPPB (Fig. S2). The XLogP3-AA value^39^ of SH-5 is -1.3, while that of NPPB is 4.1. Thus, NPPB has higher membrane permeability and shows stronger antigrowth activity against the *P*. *gingivalis* strain than SH-5. Because NPPB and SH-5 were not toxic toward *E*. *coli* at the concentrations used for the inhibition of *P*. *gingivalis* growth (Fig. 6, open circles), it is expected that the antigrowth activity of NPPB and SH-5 against *P*. *gingivalis* is due to inhibition of PgDPP11 by the common core structure of these molecules; the nitrobenzoic acid moiety can form a hydrogen bond network with Thr650 and Asn670 and can form an electrostatic interaction with Arg673 (Fig. 4b). Insensitivity of *E*. *coli* cells against NPPB and SH-5 (Fig. 6, open circles) is reasonable, because the nitrogen source of *E*. *coli* growth is ammonium ions in the medium and is independent from the peptide digestion system, in which DPPs are involved.

However, notably, inhibition of PgDPP11 cannot by itself explain the strong inhibitory effect of NPPB against the growth of *P*. *gingivalis*. Another protein, e.g., a peptide transporter or an ion channel, in *P*. *gingivalis* might be inhibited by NPPB, and the apparent antigrowth effect of NPPB could be enhanced, because some chloride ion channels (ClCs) in mammals are reported to be inhibited by NPPB (Table S1 and references therein). To determine whether a molecular target of NPPB other than DPP11 exists in *P*. *gingivalis*, we searched for orthologs of mammalian-type ClCs in *P*. *gingivalis* strain W83 and *E*. *coli* strain K12 based on the amino acid sequences of the ClCs (Table S1). No orthologs were identified in *P*. *gingivalis* strain W83, whereas ClC-ec1 in *E*. *coli* strain K12 was identified to be an ortholog of ClC-2 to ClC-7 of mammals. ClC-ec1 protein has been reported to not be inhibited by NPPB^40^, which is consistent with our result showing that NPPB was not toxic toward *E*. *coli* at the concentrations used for the inhibition of *P*. *gingivalis* growth (Fig. 6, open circles). Thus, the higher inhibitory effect of NPPB against the growth of *P*. *gingivalis* appears to be unrelated to the inhibition of ClCs and remains poorly understood. Although this point should be clarified in greater detail, such an explanation is beyond the scope of this paper.

The process of inhibitor discovery in the present study has two unique features. The first is the use of a space-grown crystal-derived high-resolution structure as the template for pharmacophore setting. We solved the crystal structure of PgDPP11 complexed with citrate and potassium ions using a space-grown crystal obtained in the Japanese experimental module “Kibo” at the ISS^41^. The high-resolution structure enabled us to unequivocally identify bound ions and water molecules in the active site of PgDPP11 and to hypothesize that the bound ions mimic the binding of an acidic amino acid, which corresponds to the preferred substrate P1 residue of PgDPP11. In general, if crystallization conditions are properly optimized, high-quality protein crystals can be obtained in space^41–43^. Typical examples obtained in the Kibo module at the ISS are atomic-resolution structural analyses of hematopoietic prostaglandin D synthase^44^ and lipocalin-type prostaglandin D synthase^45^. However, to the best of our knowledge, the application of a space-grown crystal-derived high-resolution structure for *in silico* screening has not been previously reported. This study shows the practical applicability of protein crystallization under a microgravity environment. Thus, the benefit of protein crystallization on the ISS in the present study is the improvement of resolution as compared with our previous study rather than *de novo* crystallization.

The second unique feature of the process of inhibitor discovery in the present study is the incorporation of ion and solvent molecules in the pharmacophore used. We generated a 3D pharmacophore model (Fig. 4a) that included a total of only three features, consisting of two HBA and one HBD features: the two HBA features (Fig. 4a, magenta spheres) at the centroid of the two oxygen atoms of the distal carboxy group of citrate and the position of the oxygen atom of HOH240 and one HBD feature (Fig. 4a, cyan sphere) at the position of the K^+^ ion. In most cases of pharmacophore-based *in silico* screening, pharmacophore features are set on the atoms of bound fragment molecules. In the present study, however, the size of the bound citrate molecule was too small and imperfect to mimic the substrate peptide; although the distal carboxy group of citrate corresponds to the side chain of the acidic amino acid, the citrate molecule lacks elements of the peptide main chain. Therefore, the position of the K^+^ ion was assumed to be the binding site of the positively charged N-terminus, and the bound water molecule HOH240 was interpreted as occupying the binding site of the carbonyl group of the P1 residue of the bound peptide. Because displacement/replacement of water molecules from the ligand binding site is often energetically favorable for inhibitor design^46,47^, incorporation of a water molecule in the pharmacophore model is reasonable. Therefore, we generated a pharmacophore feature at the position of the oxygen atom of HOH240 without hesitation, since the water molecule at that position is expected to be replaced by the substrate peptide. The availability of water molecules as the pharmacophore model in combination with molecular dynamics simulations has been reported in a number of studies^48–50^, and typical examples of such a procedure are the programs WaterMap^48^ and GIST^49^. The pharmacophore setting in the present study is somewhat similar to the concepts of these programs in that the setting considers the thermodynamic properties of water (and ion) molecules that solvate ligand-binding sites. However, the setting is clearly different from the already-existing procedure in that the positions of water (and ion) molecules in the present study are based on a high-resolution experimentally determined electron density map rather than a calculated model.

In conclusion, we have discovered the first nonpeptidyl inhibitor of S46 peptidases, SH-5, by fragment-based *in silico* screening. The target selectivity of the inhibitor, that is the inhibitory effects against DPP11s over DPP7s, is explained by a specific hydrogen bond network and an electrostatic interaction between the inhibitor and the active site residues of PgDPP11 that are conserved among DPP11s but not in DPP7s. Thus, discovery of the hit compound SH-5 and its lipophilic analog NPPB will be a good starting point for the design of potent and specific inhibitors of DPP11s. In addition, the space-grown crystal-derived high-resolution crystal structure was extremely useful for the pharmacophore setting of the *in silico* screening: the binding mode of the substrate peptide was assumed, and the pharmacophore was composed of only three features. This result provides a good example of the usefulness of a high-resolution crystal structure and new insights into structure-based inhibitor design.

## Methods

### Overexpression and purification of PgDPP11

A synthetic gene encoding full-length PgDPP11 (residues 1-720, UniProt accession number B2RID1), codon-optimized for expression in *E*. *coli*, was purchased from Genscript (NJ, USA). The target sequence corresponding to mature PgDPP11 (Asp22-Pro720) containing the signal peptide of DAP BIII^25,51^ from *P*. *mexicana* WO24 was amplified using PCR and cloned into the pET22b expression plasmid (Merck, Darmstadt, Germany). *E*. *coli* BL21 Gold(DE3) cells (Agilent Technologies, Santa Clara, CA, USA) transformed with the pET22b-PgDPP11 expression plasmid (Merck) were grown in TB media at 298 K. Overproduction of PgDPP11 was performed by isopropyl-β-D-thiogalactopyranoside (IPTG) induction (final concentration, 0.1 mM) at an OD_600_ of approximately 0.6. Fifteen hours after induction, the cells were harvested by centrifugation at 8,000 × g. The cells were disrupted using BugBuster protein extraction reagent (Merck). The cell extract was obtained by centrifuging the lysate at 27,000 × g for 30 min. PgDPP11 was purified by precipitation with 35 to 70% ammonium sulfate and hydrophobic column chromatography using a HiPrep 16/10 butyl column (GE Healthcare, Little Chalfont, UK). The eluate was desalted using a HiPrep 26/10 desalting column (GE Healthcare) and finally subjected to anion-exchange column chromatography using a Mono Q 5/50 GL column (GE Healthcare). The fractions containing PgDPP11 were pooled, and the buffer was exchanged with 80 mM Tris-HCl (pH 8.5) and concentrated to 10 mg/ml using a Vivaspin 20 concentrator (GE Healthcare). The protein concentration was determined using the Bradford assay (Bio-Rad Laboratories, Hercules, CA, USA) and bovine gamma-globulin ranging from 0 to 0.25 mg/ml was used as a standard. The column chromatography and other purification steps were performed at 298 K and 277 K, respectively.

### Overexpression and purification of PgDPP7, SmDPP7 and SmDPP11

Synthetic genes encoding full-length PgDPP7 (residues 1–712, UniProt accession number Q7MWU6), SmDPP7 (residues 1–720, UniProt accession number B4SLK2) and SmDPP11 (residues 1–715, UniProt accession number B4SNQ8), codon-optimized for expression in *E*. *coli*, were purchased from Genscript (NJ, USA). The target sequences corresponding to mature PgDPP7 (Asp25-Ile712), mature SmDPP7 (Ala23-Lys720) and mature SmDPP11 (Asp24-Gln715), each containing the signal peptide of DAP BIII^25,51^ from *P*. *mexicana* WO24, were amplified using PCR and cloned into the pET22b expression plasmid (Merck, Darmstadt, Germany). Overproduction and purification of PgDPP7, SmDPP7 and SmDPP11 were performed in a manner similar to the method described for PgDPP11 described above.

### Crystallization

To obtain citrate-bound PgDPP11 crystals, the samples were initially crystallized using the hanging-drop method, in which 1 μl of protein solution (10 mg/ml PgDPP11 in 80 mM Tris-HCl (pH 8.5)) was mixed with the same volume of reservoir solution (20%(m/v) PEG8000 and 0.2 M tri-potassium citrate) and incubated at 293 K. The drops were suspended over 200 μl of reservoir solution in 48-well plates. The citrate-bound crystals were also obtained using a counterdiffusion crystallization method^52^, in which 20%(m/v) PEG8000 and 0.2 M tri-potassium citrate was used as a reservoir solution, under a microgravity environment in the Japanese experimental module “Kibo” at the ISS^41^. Crystals of the PgDPP11/inhibitor complex were prepared as follows. SH-5 (2-[(2-aminoethyl)amino]-5-nitrobenzoic acid, C_9_H_11_N_3_O_4_) was purchased from Namiki Shoji Co., Ltd. (Tokyo, Japan) and was dissolved in 100% DMSO to a concentration of 10 mM. The 5-mg/ml PgDPP11 solution was mixed with the SH-5 solution at a volumetric ratio of 19:1, with a final inhibitor concentration of 0.5 mM. The working solution was crystallized using the counterdiffusion method, in which 19%(m/v) PEG8000, 0.2 M magnesium formate, 0.5 mM SH-5 and 5%(v/v) DMSO in 80 mM Tris-HCl (pH 8.5) was used as a reservoir solution.

### X-ray data collection

Crystals obtained by the counterdiffusion method under a microgravity environment in the Kibo module at the ISS were used for the present crystallographic analyses. For data collection of the citrate-bound crystals under cryogenic conditions, crystals in a capillary were directly transferred to a harvesting solution [16%(w/v) PEG8000, 0.16 M tri-potassium citrate and 20%(v/v) glycerol] for 10 seconds. Crystals were mounted in nylon loops and flash-cooled in a cold nitrogen gas stream at 100 K immediately before data collection. For data collection of the SH-5-bound crystals, 16%(m/v) PEG8000, 0.16 M magnesium formate, 0.4 mM SH-5, 4%(v/v) DMSO and 20%(v/v) glycerol in 64 mM Tris-HCl (pH 8.5) was used as a cryoprotectant. Data were collected by the rotation method at 100 K using a PILATUS-6M detector with synchrotron radiation sources on the beamline BL17A at the Photon Factory. Laue group and unit-cell parameters were determined using the XDS software package^53^. The resulting cell parameters and data collection statistics are summarized in Table 1.

### Structure determination and refinement

The initial phase determination was performed for the citrate-bound PgDPP11 using the molecular replacement method, and one protomer of ligand-free PgDPP11 (PDB code: 4Y04) was used as the search model. Cross-rotation and translation functions were calculated using the program MOLREP^54^ from the CCP4 suite^55^. Automatic model building and refinement were carried out using the programs ARP/wARP^56^ and REFMAC5^57^, and further iterative manual model building and refinement were performed using the programs Coot^58^ and REFMAC5. The stereochemistry of the model was verified using the program RAMPAGE^59^. The refined structure of the citrate-bound PgDPP11 was then used for the structural determination of the SH-5 complex by the molecular replacement method. The occupancies of inhibitor atoms of the SH-5 complex were estimated by varying the occupancies of inhibitor atoms set at 0.60 to 0.90, in increments of 0.05. The results showed that the temperature factors of SH-5 became closer to those of the surrounding atoms, without producing a positive residual peak in the |*F*o|-|*F*c| map, when the occupancies of the ligand atoms were set at 0.85. Occupancies of 0.80 or below resulted in positive residual peaks in the |*F*o|-|*F*c| map for the bound ligand atoms. After the final round of refinement, the inhibitor atoms were removed from the model. Then, the amplitude |*F*c| and phase angles calculated from the partial structure were used to generate a weighted *m*|*F*o|-*D*|*F*c| omit map^57^, where *m* is the figure of merit (approximately equal to the cosine of the phase error) and *D* is the estimate of the coordinate error in the partial structure (Fig. 2). The refinement statistics are summarized in Table 2.

### *In silico* screening

A multifilter virtual screening protocol (Fig. 3) was performed to explore candidate compounds for novel PgDPP11 inhibitors. For this purpose, the high-resolution crystal structure of PgDPP11 in complex with a citrate ion described above, derived from a space-grown crystal, was used. Upon examination of the X-ray structure, a citrate ion was found in the active site of PgDPP11. Arg673 of PgDPP11 has been reported to play an important role in the Asp/Glu specificity of the S1 subsite of PgDPP11^23,27,31^. A distal carboxy group of the citrate ion shows ionic hydrogen bonds with the side chain of Arg673 and was found to mimic the binding mode of an acidic (Asp/Glu) side chain of the P1 residue of a substrate peptide. A water molecule (HOH240) that is hydrogen bonded to the central carboxy group of the citrate is accommodated in the oxyanion hole of PgDPP11. In addition, a potassium ion (K^+^) was identified in the P2 residue-binding site of PgDPP11 and found to mimic the binding mode of the positively charged N-terminus of the P2 position of the substrate peptide. Therefore, we explored compounds that were expected to interact with PgDPP11 in a manner similar to the distal carboxy group of the citrate ion, the water molecule HOH240, and the K^+^ ion.

### *In silico* analyses of the binding mode of SH-5 with PgDPP11

All calculations were performed using Schrödinger Suite 2013-2 (Schrödinger, LLC, New York, NY, 2013). The 3D structure of SH-5 was generated using the LigPrep2.7 program, and its protonation state was predicted using the Epik2.5 program. Then, the conformational search was carried out using the ConfGen2.5 program, and the resulting conformers were used in the following docking calculations. The A-chain of the crystal structure of PgDPP11, which is a subunit in complex with the citrate ion, was used as a receptor for docking. The crystal structure was minimized using force-field OPLS 2005 through the Protein Preparation Wizard in Maestro 9.5. We removed all citrate ion, potassium ion, and H_2_O molecules except for HOH9 and HOH710 before docking calculations. As HOH9 and HOH710 form water-mediated hydrogen bond networks between citrate ion and PgDPP11 (Fig. S3), these two water molecules may be important both in allowing PgDPP11 to recognize ligands such as citrate ion and in stabilizing the complex structure. Therefore, we considered the combinations of the presence or absence of HOH9 and HOH710 to prepare a total of four receptor structures (Fig. S4). The docking calculations of SH-5 against these four receptor structures were performed using the Glide 6.0 SP mode. The box center for the “Receptor Grid Generation” protocol was set to a centroid of the citrate ion binding to PgDPP11. A van der Waals radius scaling of 1.0 was used for both protein and ligand, and the maximum number of poses per conformer was set to 5. The generated poses were ranked according to docking score to select the top 30% docked complexes. Finally, the binding free energy (ΔG_binds_) values for these top 30% complexes were estimated with the MMGBSA method using the Prime 3.3 program. We selected a docked complex with the lowest ΔG_bind_ as the interaction model (Fig. 4b). To test the docking procedure, we applied the procedure for the binding of citrate ion with PgDPP11. The result is shown in Fig. S5. The resulting model, i.e., the top-ranked pose, reproduced all the interactions between citrate ion and PgDPP11 and the water-mediated hydrogen bonds between citrate ion and PgDPP11 through HOH9 and HOH710 observed in the crystal structure. The positional and conformational root mean square deviations between the crystal and docked poses of citrate ion were 0.55 and 0.27 Å, respectively. This result suggested that the procedure is adequate for the generation of a reliable model of the binding between SH-5 and PgDPP11.

### Evaluation of the inhibitory effects of *in silico* candidate compounds against PgDPP11, PgDPP7, SmDPP7 and SmDPP11

Thirteen of fifteen final candidate compounds from the *in silico* screening described above were purchased from Namiki Shoji Co., Ltd. (Tokyo, Japan). Half-maximal inhibitory concentration (IC_50_) values were determined by fitting to a sigmoidal curve (4-parameter logistic curve) using ImageJ^60^ Competitive inhibition rates were measured using different concentrations of each inhibitor in a reaction buffer containing 50 mM sodium phosphate buffer (pH 8.0), 5 mM EDTA and 0.005% Tween 20 at 298 K for 20 min and using 100 µM Leu-Asp-MCA (4-methylcoumaryl-7-amide) as the substrate for DPP11s. For DPP7s, 100 µM Met-Leu-MCA was used as the substrate. The concentrations of PgDPP11, PgDPP7, SmDPP11 and SmDPP7 were 1, 10, 0.5 and 5 nM, respectively. The inhibitor concentrations were 1000, 500, 250, 125, 62.5, 31.3 and 15.6 µM. Standard deviations were calculated from three independent experiments. Fluorescence intensity was measured with excitation at 355 nm and emission at 460 nm using an Infinite 200 PRO microplate reader. Inhibition constants (*K*_i_) were calculated using the Cheng-Prusoff equation^61^. Inhibitory effects of the thirteen compounds against PgDPP11 and those of SH-5 and NPPB against four DPPs are summarized in Fig. 5 and Table 3, respectively

### Biological evaluation

Antibacterial assays against *P*. *gingivalis* strain W83 and *E*. *coli* strain K12 were performed as previously described^62,63^. *P*. *gingivalis* cells were cultured in anaerobic bacterium culture medium (ABCM, Eiken Chemical, Tokyo, Japan) containing 0.001% menadione under anaerobic conditions at 37 °C for 48 h. *E*. *coli* cells were grown in M9 medium with 1% glucose with shaking at 37 °C for 24 h. All compounds were dissolved in DMSO at stock concentrations of 100 mM. Further dilutions were prepared in complete culture medium so that the final concentrations of the solvent did not interfere with bacterial growth. Growth of *P*. *gingivalis* and *E*. *coli* with compounds was determined as the optical density at 600 nm after incubation. The results are shown in Fig. 6.

### Graphical programs

Figures 1, 2 and 4 were produced using the programs UCSF Chimera^64^ and Adobe Illustrator (Adobe Systems Inc., San Jose, CA, USA). Figures 3, 5, 6 and 7 were produced using Adobe Illustrator. Figures S1 and S2 were produced using the program Biovia Draw (Dassault Systems, France). Figures S3–S5 were produced using the program Maestro (Schrödinger, LLC, New York).

### Accession codes

Atomic coordinates for the reported structures have been deposited in the Protein Data Bank under accession codes 6JTB (citrate complex) and 6JTC (SH-5 complex).

## Supplementary information

Supplementary Information
