# Supplementary material for: Fragment-based discovery of the first nonpeptidyl inhibitor of an S46 family peptidase
Source: Sci Rep. 2019 Sep 19;9:13587. doi: 10.1038/s41598-019-49984-3 (PMC6753110; doi:10.1038/s41598-019-49984-3)
Supplement: Supplementary file 1 — Supplementary Information [file 41598_2019_49984_MOESM1_ESM.pdf]

SUBJECT AREAS:

CHEMICAL BIOLOGY, DRUG DISCOVERY, STRUCTURAL BIOLOGY

[*]Correspondence and requests for materials should be addressed to
N.T. (ntanaka@pharm.showa-u.ac.jp)

Fragment-based discovery of the first nonpeptidyl inhibitor of an S46 family peptidase.

Yasumitsu Sakamoto[1], Yoshiyuki Suzuki[2,3], Akihiro Nakamura[2], Yurie Watanabe[4], Mizuki Sekiya[1], Saori Roppongi[1], Chisato Kushibiki[1], Ippei Iizuka[1], Osamu Tani[5], Hitoshi Sakashita[5], Koji Inaka[6], Hiroaki Tanaka[7], Mitsugu Yamada[8], Kazunori Ohta[8], Nobuyuki Honma[2], Yosuke Shida[2], Wataru Ogasawara[2], Mayumi Nakanishi-Matsui[1], Takamasa Nonaka[1], Hiroaki Gouda[4], and Nobutada Tanaka[4,9,*]

[1]School of Pharmacy, Iwate Medical University, 1-1-1 Idaidori, Yahaba, Iwate 028-3694, Japan; [2]Department of Bioengineering, Nagaoka University of Technology, 1603-1 Kamitomioka, Nagaoka, Niigata 940-2188, Japan; [3]National College of Technology, Nagaoka College, 888 Nishikatakai, Nagaoka, Niigata 940-8532, Japan; [4]School of Pharmacy, Showa University, 1-5-8 Hatanodai, Shinagawa-ku, Tokyo 142-8555, Japan; [5]Biomedical Research Institute, National Institute of Advanced Industrial Science and Technology (AIST), 1-1-1 Higashi, Tsukuba, Ibaraki 305-8566, Japan; [6]Maruwa Foods and Biosciences Inc., 170-1 Tsutsui-cho, Yamatokoriyama, Nara 639-1123, Japan; [9]Confocal Science Inc., 2-12-2 Iwamoto-cho, Chiyoda-ku, Tokyo 101-0032, Japan; [8]Japan Aerospace Exploration Agency (JAXA), 2-1-1 Sengen, Tsukuba, Ibaraki 305-8505, Japan; [9]Center for Molecular Analysis, Showa University, 1-5-8 Hatanodai, Shinagawa-ku, Tokyo 142-8555, Japan

Supplementary information includes: Figures S1-S5 and Table S1.

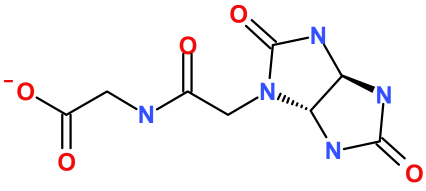

SH-1
(NS-000369443)

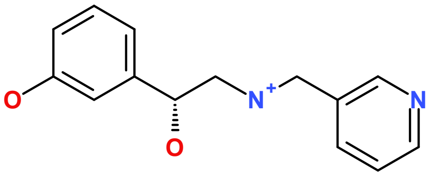

SH-2
(NS-013008538)

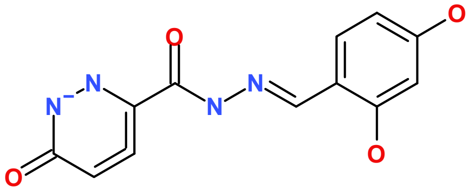

SH-3
(NS-006153306)

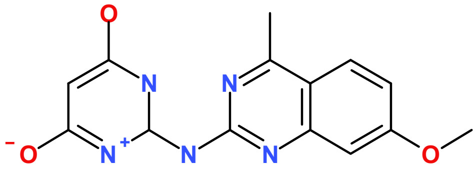

SH-4
(NS-005958011)

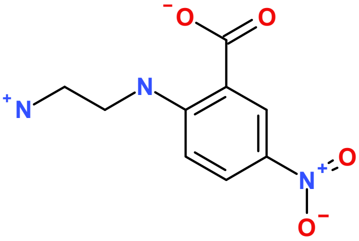

SH-5
(NS-006172452)

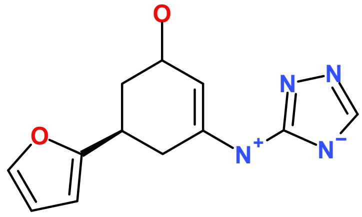

SH-6
(NS-012335443)

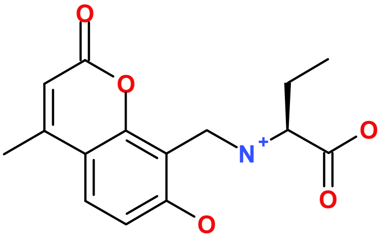

SH-7
(NS-006029553)

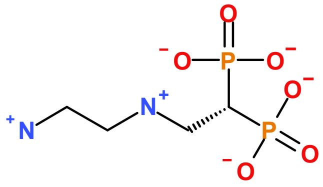

SH-8
(NS-014544273)

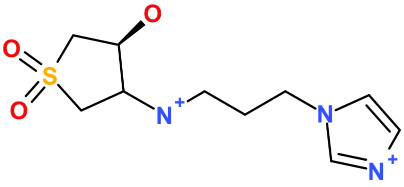

SH-9
(NS-006808995)

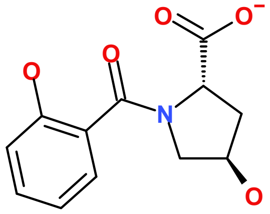

SH-10
(NS-000528873)

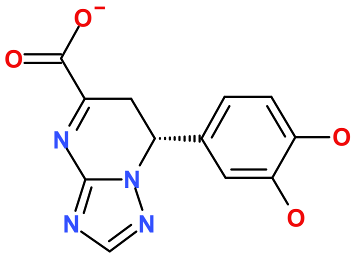

SH-11
(NS-012518617)

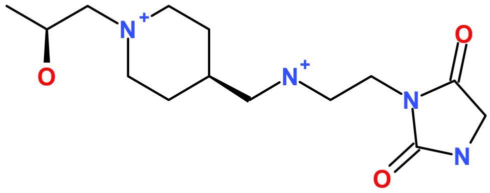

SH-12
(NS-000825843)

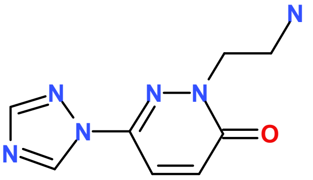

SH-13
(NS-019767473)

Fig. S1. Thirteen candidate compounds obtained by the third-stage screening.
Aliases in this paper (SH-1 to SH-13) are shown below the compounds
and vendor IDs are shown in parentheses (NS stands for Namiki Shoji).

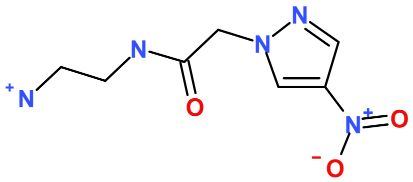

SH-5_1
(NS-000379699)

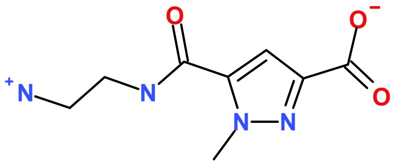

SH-5_2
(NS-006541971)

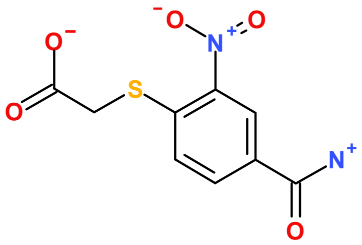

SH-5_3
(NS-000466920)

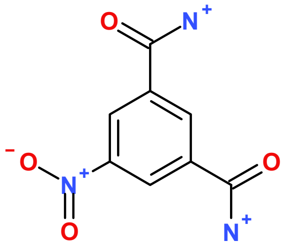

SH-5_4
(NS-005896597)

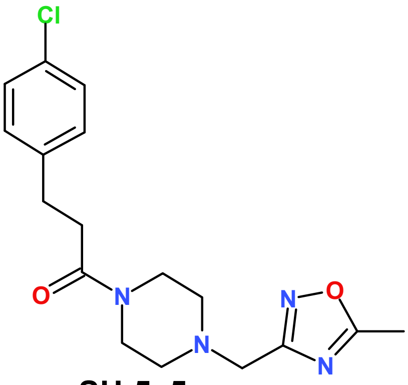

SH-5_5
(NS-019447780)

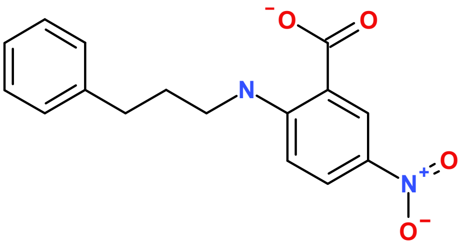

NPPB
(Merck-N4779)

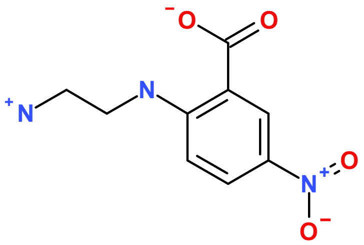

SH-5
(NS-006172452)

Fig. S2. Five additional candidate compounds based on 2D structural fingerprints analysis and a lipophilic analog of SH-5, NPPB (5-nitro-2-(3-phenylpropylamino)benzoic acid). Aliases in this paper (SH-5_1 to SH-5_5) are shown below the compounds, and vendor IDs are shown in parentheses (NS stands for Namiki Shoji). SH-5 is shown for reference.

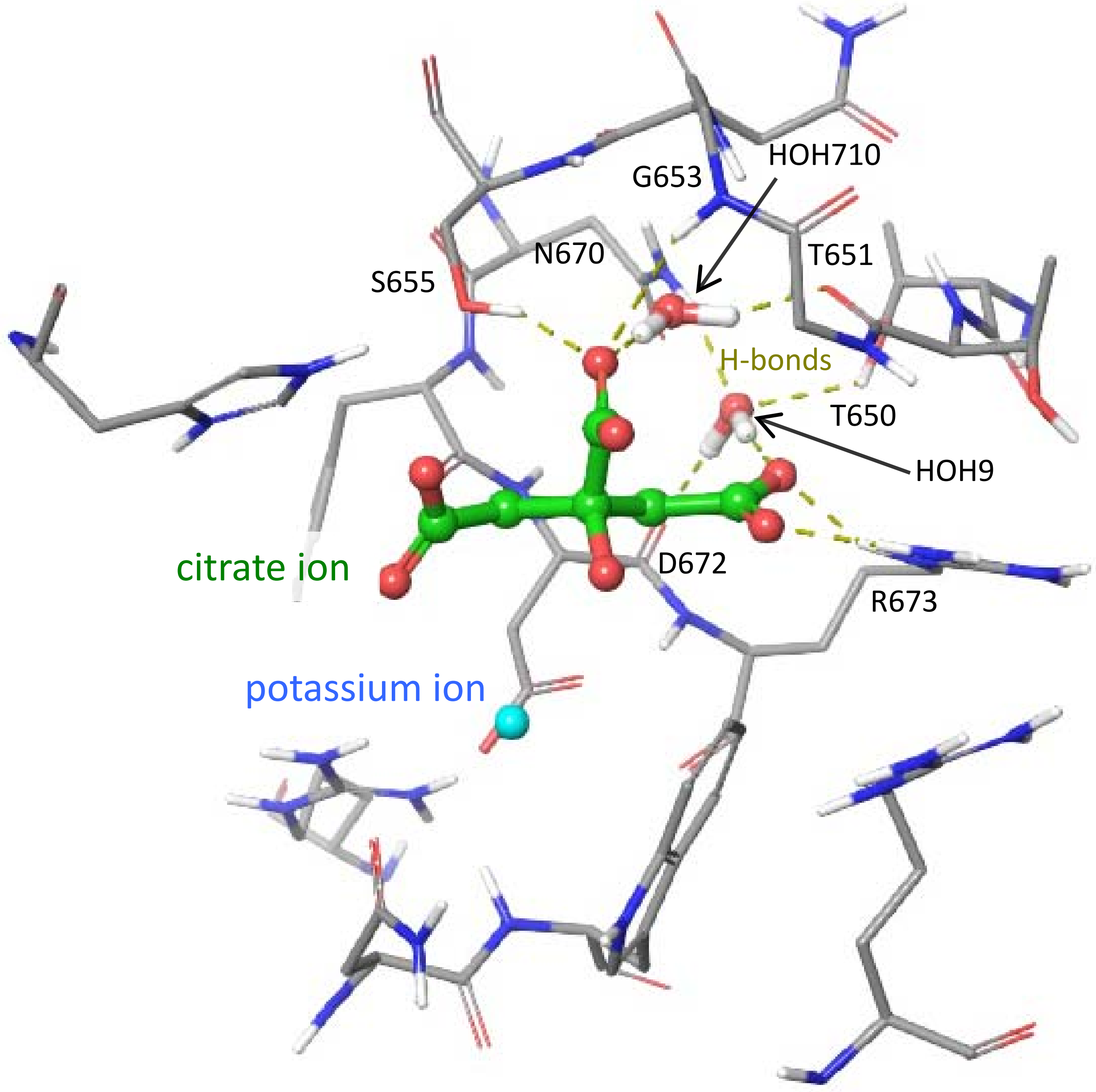

Fig. S3. A water-mediated hydrogen bond network between the bound citrate ion and the S1 subsite of PgDPP11.

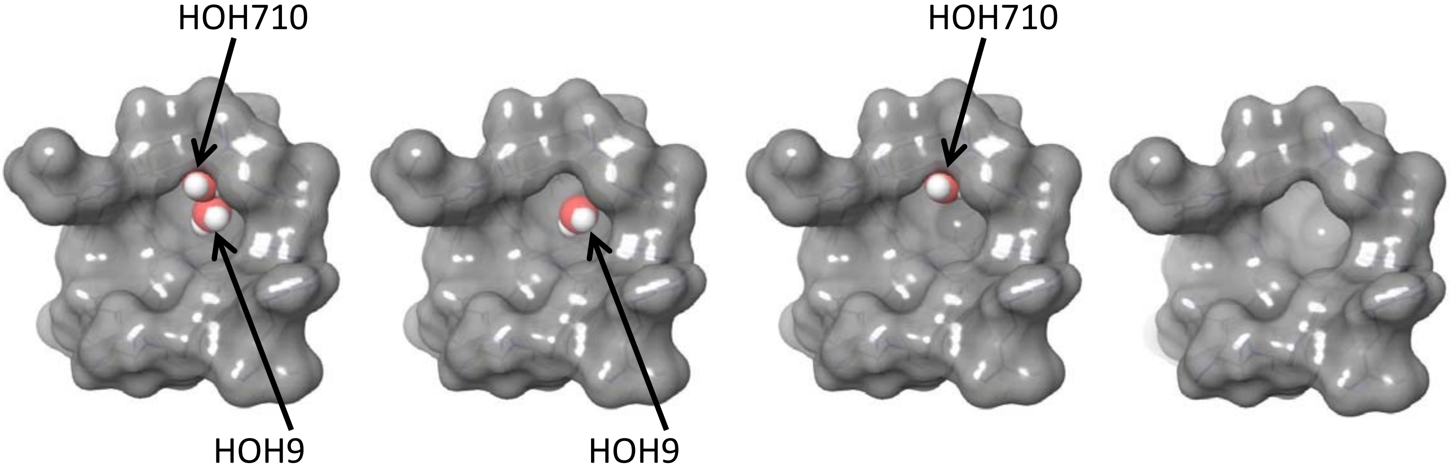

Fig. S4. Four receptor structures to examine the importance of water molecules in the S1 subsite of PgDPP11 for docking calculations.

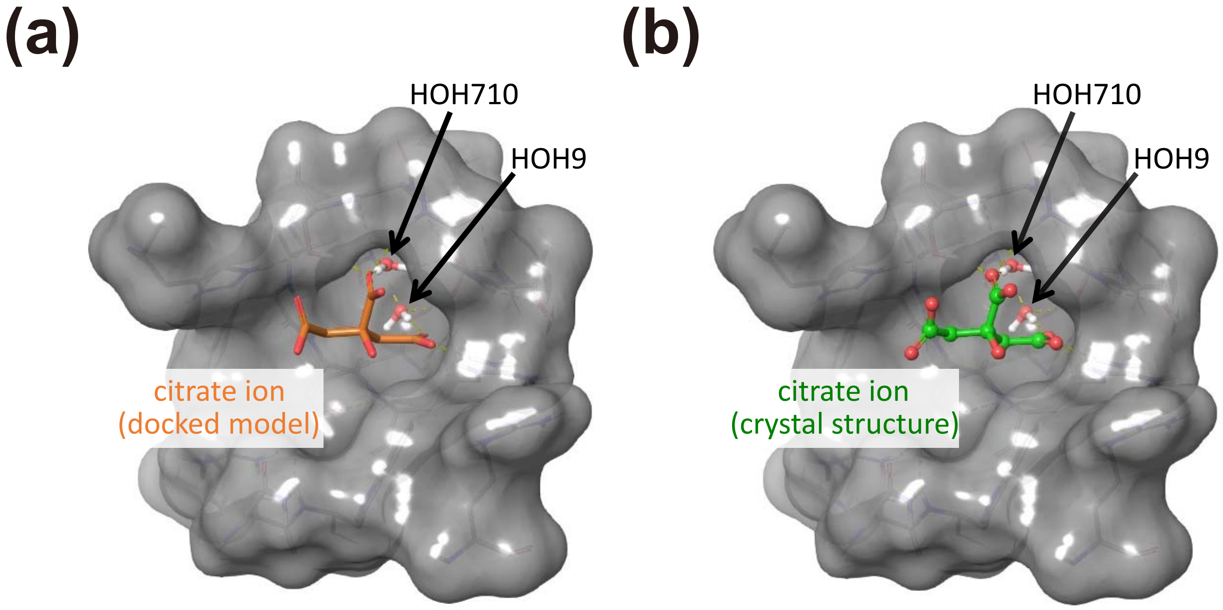

Fig. S5. Redocking of a citrate ion in the S1 subsite of PgDPP11 to verify the docking procedure.

(**A**) Docked model. (**B**) Crystal structure.

Table S1 | Mammalian chloride ion channels that are inhibited by NPPB.

Chloride ion channel	Species	UniProt ID	Reference
ClC-2	Rat	P35525	[1]
ClC-5	Mouse	Q9WVD4	[2]
ClC-6	Human	P51797	[3]
ClC-7	Human	P51798	[4]
ANO-1	Mouse	Q8BHY3	[5]
ANO-2	Mouse	Q8CFW1	[6]
BEST-1	Mouse	O88870	[7]
CFTR	Xenopus	P26363	[8]

[1] H. Xiong et al., "ClC-2 activation modulates regulatory volume decrease," *J. Membr. Biol.* **167**, 215–221 (1999).

[2] M. Hara-Chikuma, Y. Wang, S. E. Guggino, W. B. Guggino, and A. S. Verkman, "Impaired acidification in early endosomes of ClC-5 deficient proximal tubule," *Biochem. Biophys. Res. Commun.* **329**, 941–946 (2005).

[3] G. Buyse et al., "Expression of human pI(Cln) and ClC-6 in Xenopus oocytes induces an identical endogenous chloride conductance," *J. Biol. Chem.* **272**, 3615–3621 (1997).

[4] H. Kajiya, F. Okamoto, K. Ohgi, A. Nakao, H. Fukushima, and K. Okabe, "Characteristics of ClC7 Cl^- channels and their inhibition in mutant (G215R) associated with autosomal dominant osteopetrosis type II in native osteoclasts and hClcn7 gene-expressing cells," *Pflugers Arch. Eur. J. Physiol.* **458**, 1049–1059 (2009).

[5] R. D. Singh et al., "Ano1, a Ca^{2+}-activated Cl-channel, coordinates contractility in mouse intestine by Ca^{2+}transient coordination between interstitial cells of Cajal," *J. Physiol.* **592**, 4051–4068 (2014).

[6] K. Bernstein et al., "Calcium-activated chloride channels anoctamin 1 and 2 promote murine uterine smooth muscle contractility," *Am. J. Obstet. Gynecol.* **211**, 688.e1-688.e10 (2014).

[7] H. Park et al., "High glutamate permeability and distal localization of Best1 channel in CA1 hippocampal astrocyte," *Mol. Brain* **6**, 1–9 (2013).

[8] Z. R. Zhang, S. Zeltwanger, and N. A. McCarty, "Direct comparison of NPPB and DPC as probes of CFTR expressed in Xenopus oocytes," *J. Membr. Biol.* **175**, 35–52 (2000).
